# Supplementary material for: Scaling behavior of electron decoherence in a graphene Mach-Zehnder interferometer
Source: Nat Commun. 2022 Sep 17;13:5473. doi: 10.1038/s41467-022-33078-2 (PMC9482640; doi:10.1038/s41467-022-33078-2)
Supplement: Supplementary file 1 — Supplementary Information [file 41467_2022_33078_MOESM1_ESM.pdf]

Supplementary information: Scaling behavior of decoherence in a graphene Mach Zehnder interferometer

M. Jo*,[1] June-Young M. Lee*,[2] A. Assouline,[1] P. Brasseur,[1] K. Watanabe,[3] T. Taniguchi,[3] P. Roche,[1] D.C. Glattli,[1] N. Kumada,[4] F.D. Parmentier,[1] H.-S. Sim†,[2] and P. Roulleau†[1]

[1]*SPEC, CEA, CNRS, Université Paris-Saclay, CEA Saclay, 91191 Gif sur Yvette Cedex France*

[2]*Department of Physics, Korea Advanced Institute of Science and Technology, Daejeon 34141, Korea*

[3]*National Institute for Materials Science, 1-1 Namiki, Tsukuba, 305-0044, Japan*

[4]*NTT Basic Research Laboratories, NTT Corporation, 3-1 Morinosato-Wakamiya, Atsugi 243-0198, Japan*

(Dated: August 27, 2022)

CONTENTS

I. Sample fabrication 3

II. Sample geometry 3

III. Thickness of the BN, role of the dielectric and comparison with GaAs interferometers 5

IV. ($\nu_L = 1$,$\nu_R = -2$) and ($\nu_L = 2$,$\nu_R = -2$) configuration 6

V. Tuning the length of the interferometer 8

VI. Determination of the visibility and error bars 11

VII. Noise setup 12

A. Resonant RLC tank circuit 12

B. Amplification chain 12

C. Base sample temperature 12

VIII. Shot noise of the beam splitters 14

IX. Measurement of the electronic temperature 14

X. 1/f noise and decoherence 16

XI. Theoretical models 17

A. Capacitive intra-edge interaction 17

B. Dependence of lobe pattern on beam splitting probability and comparison with the intra-channel interaction model 19

C. Short-range inter-edge interaction 21

XII. Aharonov-Bohm oscillations as a function of the temperature 23

Supplementary References 23

References 23

I. SAMPLE FABRICATION

Graphene, hBN, and graphite flakes were mechanically exfoliated onto the surface of SiO2/Si using the conventional scotch tape method. Graphene and graphite flakes were prepared by exfoliating bulk graphite from NGS Naturgraphit GmbH and hBN flakes were obtained by exfoliating bulk crystals from NIMS. By optical microscope, single-layer graphene was chosen. And this has been further confirmed with Raman spectroscopy after successful encapsulation with hBNs. By doing this we can avoid any spurious signature in the Raman spectrum of graphene [1]. All the crystals were carefully checked with optical microscopy and noncontact mode AFM for any defect, contamination and inhomogeneity in thickness before stacking. Using the van der Waals (vdW) dry transfer method, graphene was encapsulated in hBNs [2]. Then this BN/graphene/BN stack was transferred on a flake of graphite (9nm), which is used as a back gate. Top BN=27nm, bottom BN = 33nm. The finished stack was annealed in vacuum at 350°C to enhance the quality of the sample and then it was checked with noncontact mode AFM to locate bubbles and defects. Poly(methyl methacrylate) (PMMA) pattern defined by e-beam lithography was used as a mask for O2/CHF3 reactive ion etch (RIE) to expose the 1D edge of graphene. And using the same PMMA layer as a liftoff mask, edge contacts were defined by e-beam evaporating Cr/Au=10/30nm while rotating the sample using a tilted rotation stage. We etched the graphene channel into desired mesa geometry by using the same RIE procedure with PMMA resist mask. PMMA resist was cleanly removed in hot Acetone later. To prevent any possible leakage between the exposed graphene edge and additional gates, 10nm hBN was picked up and dry transferred on top of the sample. When removing PPC, annealing was not used this time to avoid any possible degradation of ohmic contacts. Melted PPC was cleaned with hot Acetone. Side gates were defined by evaporating Cr/Au=10/30nm. An additional 15nm BN was picked up and dry transferred on top of the sample like described before. Then finally top gates were defined by evaporating Cr/Au=10/40nm (in Supplementary Figure S1).

II. SAMPLE GEOMETRY

The sample fabricated is composed of two top gates (in Supplementary Figure S2a). Only the right top gate circled by a red dashed line have been used in this study. As shown in the schematic of the used part of the sample (in Supplementary Figure S2b), two PN junctions are generated by the top gate : one on the right without side gates plays the role of a test sample, and the other

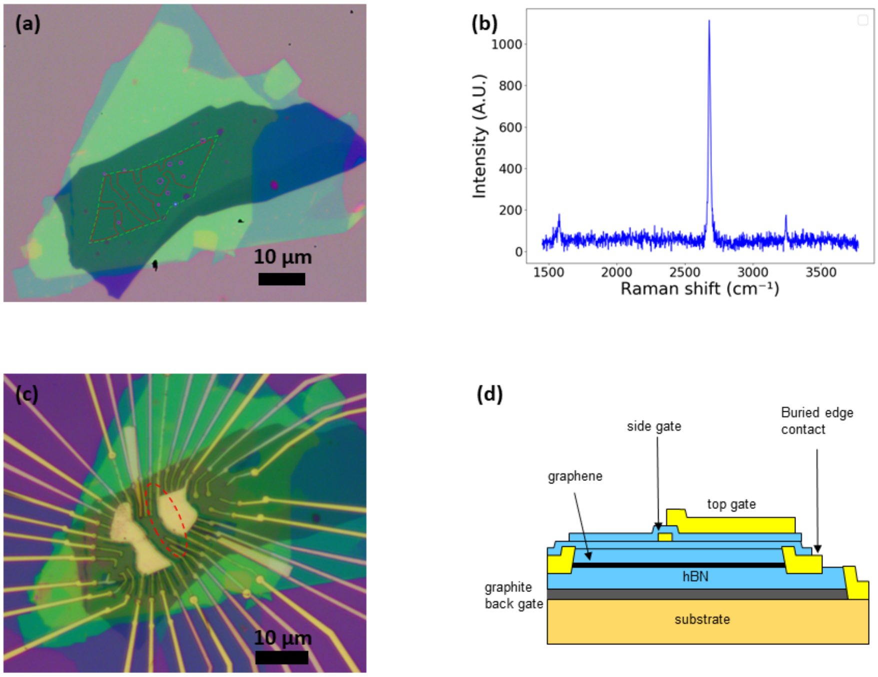

Supplementary Figure S 1. **Sample description.** (a) Optical image of BN/graphene/BN/graphite stack. The green dashed line indicates the edge of graphene. Red line indicates graphene mesa which would be defined by subsequent RIE etch. Purple circles indicate bubbles in the stack which later would be eliminated by ohmic contacts fabrication or mesa etching. (b) Raman spectroscopy of encapsulated single-layer graphene. This data was taken after successful encapsulation of graphene with top and bottom hBN but before adding additional graphite. We could minimize the spurious substrate effect and also avoid overlapping with the spectrum of graphite underneath. (c) Optical image of a finalized device. The dashed red line indicates one PN-junction, which defined by applying gate voltage from the graphite back gate and metallic top gate. (d) Cut plane view of the sample, equivalent of dashed red line section in (c)

one on the left with side gates correspond to the valley splitter sample. Precise dimensions of the valley splitter sample are presented in Supplementary Figure S2c. The width of the graphene ribbon is $1.0\mu m$. The side gates are rectangles of length $0.20\mu m$ and width $0.5\mu m$ separated by distance $0.6\mu m$. Half of the side gates is below the top gate, this implies that the PN junction meets the side gates borders in their middle at a distance $0.25\mu m$ of the two other borders of each side gate. Electrons propagate along the edge of the side gate over a length L_s=450 nm. The total PN interface length is $2L_s+0.6=1.5$ μm as mentioned in the main text.

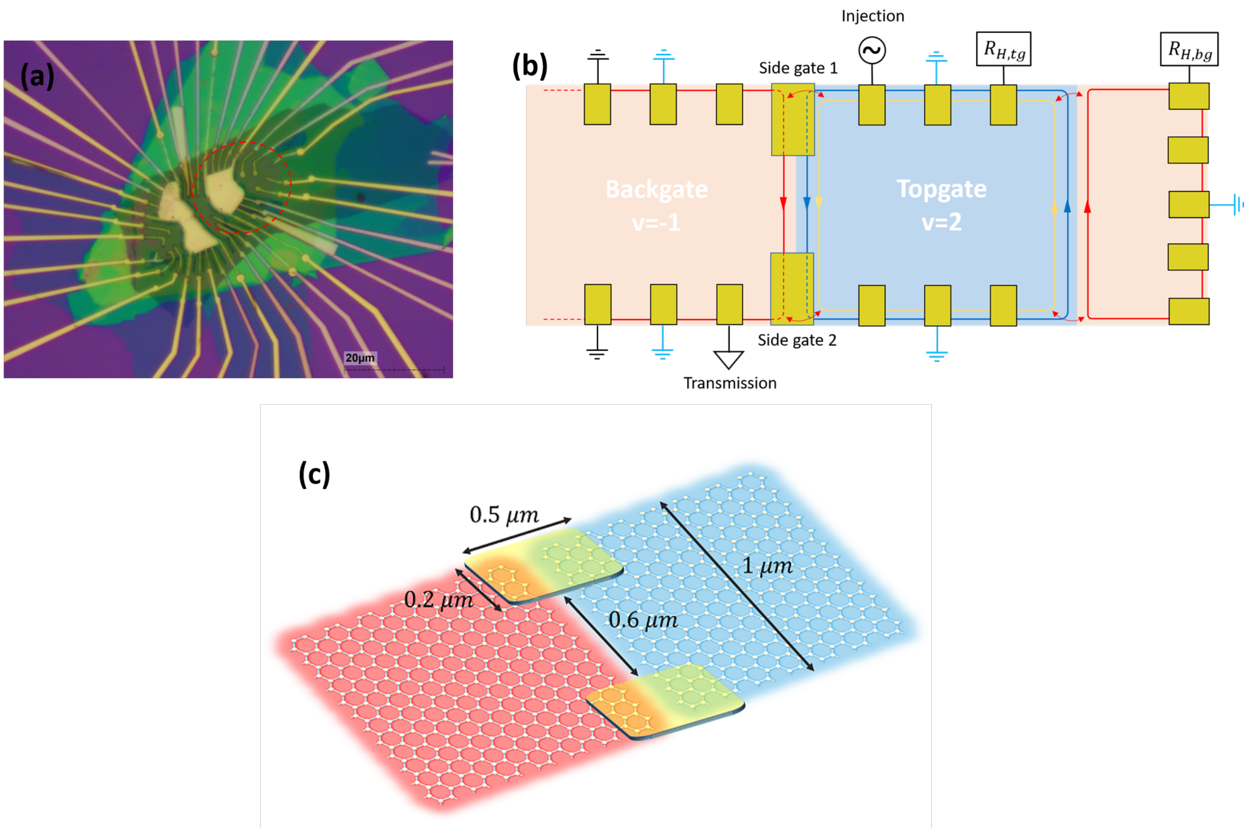

Supplementary Figure S 2. **Sample geometry.** (a) Optical micrograph of the finalized sample composed of two top gates (corresponding to the two golden plates in the center). The top gate used in this study is circled by a red dashed line. Two PN junctions are generated by the top gate : one on the right without side gates plays the role of a test sample, and another one on the left with side gates correspond to the valley splitter sample. (b) Schematic of the used part of the sample (circled by the red dashed line in (a)). The n-doped region (blue) is set at filling factor $\nu = 2$. The p-doped region under the top gate(red) is at $\nu = -1$. On the left junction, current was injected from the top right contact, and the transmitted current was measured through the bottom left contact. Note that we could not measure the reflection as the bottom right contact is not working. Light blue grounds correspond to cold grounds. The two-point Hall resistance was measured under the top gate (denoted $R_{H,tg}$) and outside (denoted $R_{H,bg}$). (c) Characteristic dimensions of the valley splitter sample : the sample width 1.0μm, the distance between the side gates 0.6μm, and a total side gate length L_s=450nm

III. THICKNESS OF THE BN, ROLE OF THE DIELECTRIC AND COMPARISON WITH GAAS INTERFEROMETERS

The bottom hBN thikness is 33nm while the top one is 27nm. An additional 10nm hBN layer is deposited to avoid any possible leakage between the exposed graphene and additional gates. On top of the side gates, a hBN layer of 15nm is deposited what makes in total 52nm of hBN (see in

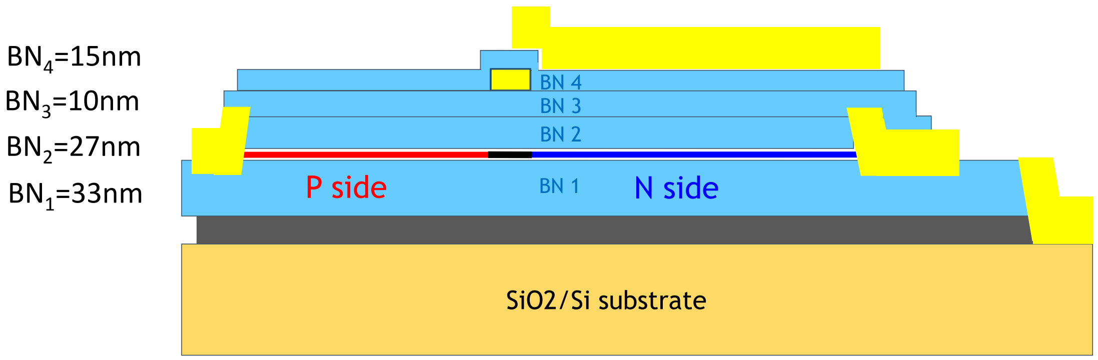

Supplementary Figure S 3. **Thickness of the BN.** (**A**) Schematic representation of the PN junction with the different BN thickness.

Supplementary Figure S3).

As shown in a recent theoretical paper [7], the distance between the interfering edge states W is essentially determined by the distance between the graphene layer and the gates d with W $\propto$ d. In GaAs, the electron gas is always deeper than 100nm leading to another interaction regime where inter-channel interactions dominate the intra-channel interactions. The role of the dielectric is also worth discussing. The dielectric constant of 10 layers of hBN is around 5 while it is 12 for GaAs. To efficiently screen Coulomb interactions in graphene, it has been shown that the considerable dielectric constant of a strontium titanate substrate ($\sim$10000) was necessary [3]. We can therefore reasonably assume that the role of the dielectric is negligible in the present case.

IV. ($\nu_L = 1$,$\nu_R = -2$) AND ($\nu_L = 2$,$\nu_R = -2$) CONFIGURATION

We check experimentally that the injected current is equal to the sum of the reflected and transmitted current through the PN junction with the side gates (device described in the main text, see in Supplementary Figure S4a). In the left region the Landau-level filling factor is $\nu_{\mathrm{L}} = 1$ and one channel circulates counterclockwise, while in the right region $\nu_{\mathrm{R}} = -2$ and two channels of the opposite spin ($\uparrow$, $\downarrow$) circulates clockwise. As shown in Supplementary Figure S4b, transmitted current in red is in phase opposition with the reflected current (in blue). Total current (in black) is conserved. Note that the AB periodicity of B=12mT (Supplementary Figure S4b) is two times smaller than described in the main text. In the main text, ($\nu_{\mathrm{L}}, \nu_{\mathrm{R}}$)=(-1,+2) while here

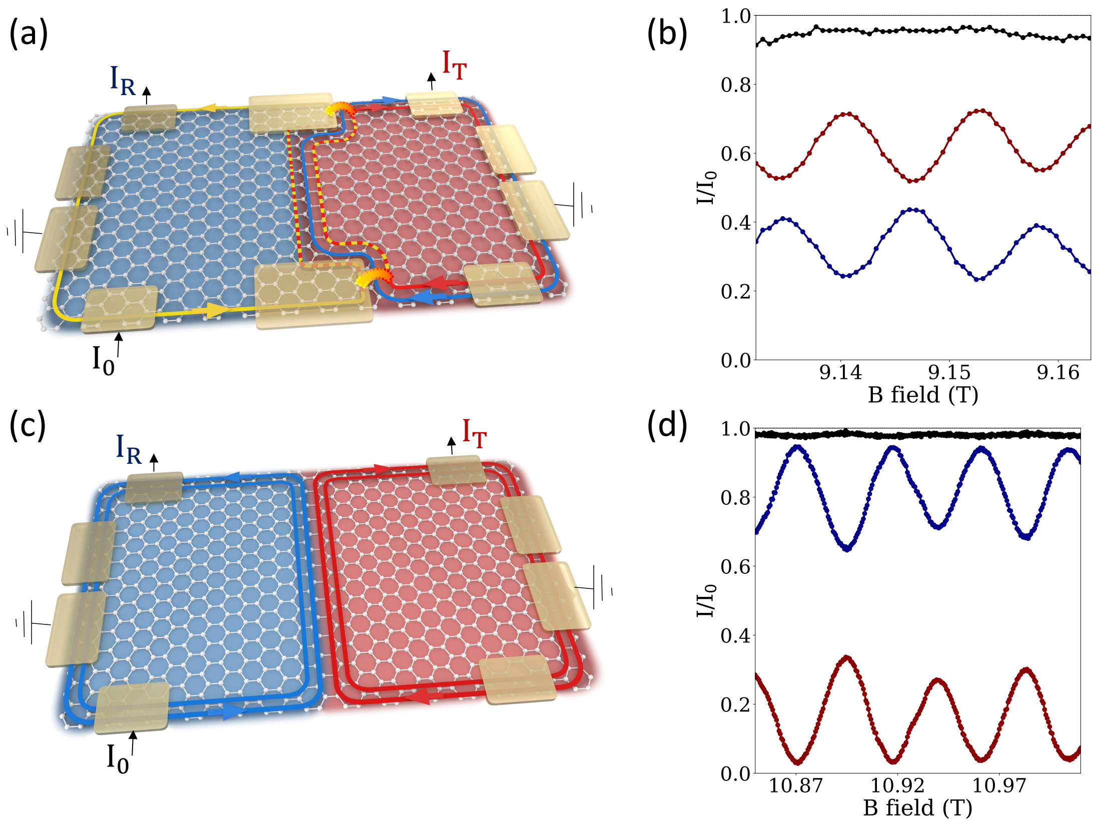

Supplementary Figure S 4. **Aharonov-Bohm oscillations as a function of the pn junction configuration** (a) Schematics representation of an interferometer with side gates (yellow). A current I_0 is injected on the n side (blue) at filling factor $\nu_L = 1$. After its transfer through the pn junction, we measure the reflected, I_R, and transmitted, I_T, current with two ohmic contacts (grey). Additional ohmic contacts are used as ground sinks. (b) Transmitted (red) and reflected (blue) current normalized by the injected current I_0, as a function of magnetic field B. These current oscillate with opposite phase and their sum (black) is conserved. (c) Schematics representation of an interferometer without side gate. A current I_0 is injected on the n side (blue) at filling factor $\nu_L = 2$. (d) Transmitted (red) and reflected (blue) current normalized by the injected current I_0, as a function of magnetic field B. These currents oscillate with opposite phase and their sum (black) is conserved.

$(\nu_\mathrm{L}, \nu_\mathrm{R})$=(+1,-2). Supplementary Figure S4c shows transmitted and reflected current for the test sample without side gates at $(\nu_\mathrm{L}, \nu_\mathrm{R})$=(+2,-2) (test sample). Total current (in black) is conserved. The AB periodicity is larger, in Supplementary Figure S4d, mostly because the total length of the interferometer, L=1μm, is smaller, due to the absence of side gates.

V. TUNING THE LENGTH OF THE INTERFEROMETER

By tuning the filling factor below both the top and bottom side gates to $\nu \leq -1$, we have two valley splitters in series defining a valley interferometer (Supplementary Figure S5A). To change the Aharonov-Bohm phase $\phi_{\mathrm{AB}} = 2\pi BA/\Phi_0$, $\Phi_0 = h/e$ being the flux quantum, we can either sweep the magnetic field or the applied voltage V_1 on the top side gate. We observe the oscillation of the transmission probability $T_{\mathrm{MZ}} = I_{\mathrm{T}}/(I_0/2)$ of the PN junction (I_0 the injected current and I_T the transmitted one (see in Supplementary Figure S5B)) with period ΔB = 20mT leading to a spatial separation of 138 nm between the two interface channels, given the length 1.5 μm of the PN interface. Valley splitting occurs at the intersections between the physical edge of graphene and the PN interface where atomic defects enable to scatter from one valley to the other. By setting $\nu_1 = 0$ below the top side gate and $\nu_2 = 0$ below the bottom side gate, we expect that no valley splitting occurs and all the injected current is reflected (see in Supplementary Figure S5C). Indeed, for negative gate voltages V1 ($\nu_1 < 0$) on the top side gate and negative gate voltages V2 (ν_2 <0) on the bottom side gate, T_{MZ} oscillates. For positive V1 ($\nu_1 \geq 0$) and V2 ($\nu_2 \geq 0$), interference pattern is completely washed out and T_{MZ}=0. Note the presence of a V2 gate voltage value (highlighted by the yellow arrow) where the MZI transmission is not zero even for $\nu_1 = 0$ and $\nu_2 = 0$. This indicates the presence of an intervalley scattering site that would not be localized along the physical edge of the graphene. We discuss this possibility in the following.

Supplementary Figure S6A depicts the MZI configuration for another set of top gate and back gate voltages where the MZI transmission can be finite even for $\nu_1 = 0$ and $\nu_2 = 0$. At $\nu_1 < 0$ below the top side gate and a fixed value of V_2 corresponding to $\nu_2 < 0$ below the bottom side gate, T_{MZ} oscillates with B and V_1 (in Supplementary Figure S6B). Above a certain value of V_1 (indicated by the dashed line in the Supplementary figure) and ν_1=0, the oscillations are suppressed. By now setting V_1 to a fix value (corresponding to $\nu_1 < 0$) and sweeping V_2, Supplementary Figure S6C shows an unusual V_2 dependence of the oscillations. For $\nu_2 < 1$, we observe two sets of oscillations: on top of the fast oscillations in V_2 we observe oscillations in B that hardly depend on V_2. Surprisingly these latter are not suppressed at ν_2=0, while the fast oscillations in V_2 disappear. We attribute these robust oscillations to the formation of a smaller interferometer where the top valley splitter is defined at the intersection between the top side gate and the physical edge, and the bottom valley splitter at the first intersection between the PN interface and the bottom side gate (in Supplementary Figure S6A). The period in B, $\Delta B \sim 34.5$ mT, leads to a spatial separation of 114 nm between the two interface channels, given the length 1.05 μm of the PN interface. We

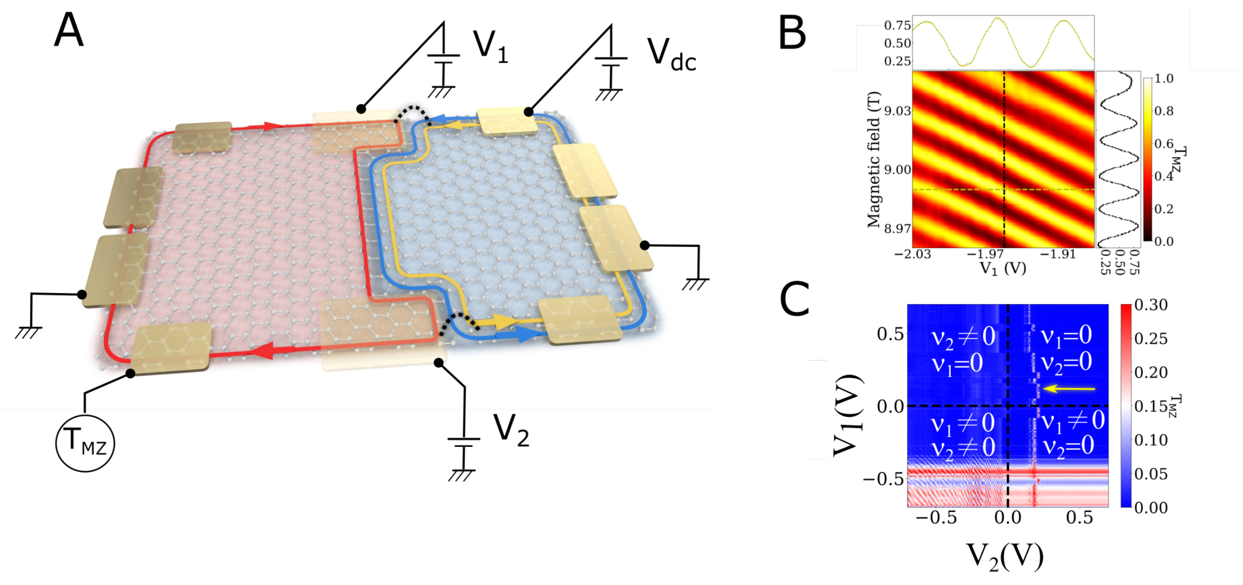

Supplementary Figure S 5. **Long length interferometer.** (**A**) Schematic representation of the PN junction. N region is depicted in blue, the P one in pink. Electrons are injected from the upper right ohmic contact (defining an injected current I_0) and transmitted current I_T is measured at the lower left contact. Buried ohmic contacts enable to simultaneously tune the filling factor by measuring the two-points Hall resistance on both sides of the junction. (**B**) Valley-interferometer transmission T_{MZ} for $\nu_1 \leq -1$ and $\nu_2 \leq -1$ as a function of B and V_1 with $T_1 = T_2 \sim 1/2$. (**C**) Valley-interferometer transmission T_{MZ} as a function of V_2 and V_1. Transition between $\nu_1 = \nu_2 = -1$ and $\nu_1 = \nu_2 = 0$ occurs for $V_1 \sim V_2 \sim 0$. The yellow arrow indicates the presence of an intervalley scattering site even for $\nu_1 = 0$ and $\nu_2 = 0$.

can also form a 1.05 μm length interferometer where the top valley splitter is defined along $\nu = 0$ in the bulk and the bottom valley splitter along the physical edge.

Following the same reasoning, we can find another set of gate voltages where both valley splitters occur along $\nu = 0$ in the bulk (in Supplementary Figure S7A). In Supplementary Figure S7B and S7C, T_{MZ} oscillations hardly depend on both V_1 and V_2. From the magnetic field periodicity $\Delta B_{MZ} \sim 81$ mT, we extract a spatial separation of 81 nm between the two interface channels, given the length 0.62 μm of the PN interface. This is the smallest electronic Mach Zehnder ever reported.

We explain the formation of the smaller interferometers by the fact that mixing can occur along an electrostatically defined edge (that is, at the edges of the regions set to $\nu = 0$ by the side gates) if there is local disorder, for instance atomic-scale defects in the bulk of graphene. As it turns out, recent STM experiments realized on the same source of graphene as ours (NGS graphenium flakes) have put into light the presence of bulk defects with rather high densities (typically 10 defects in a

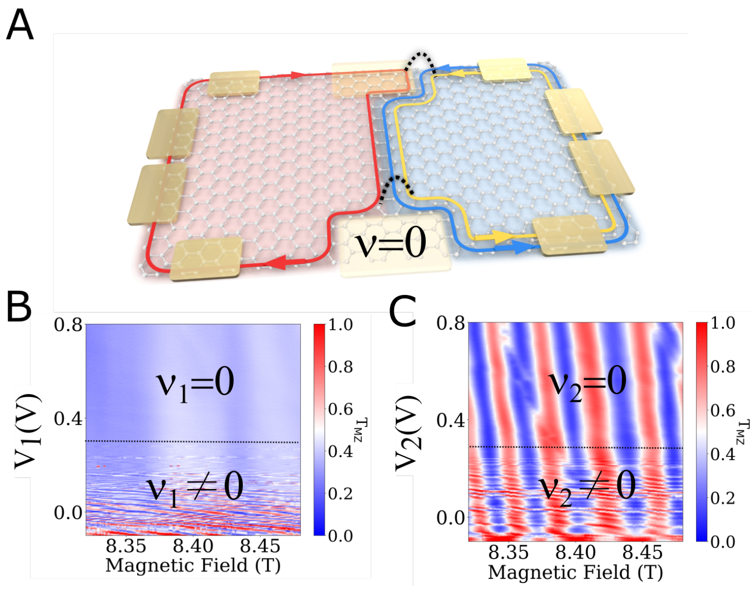

Supplementary Figure S 6. **Medium length interferometer.** (**A**) Schematic representation of the intermediate size interferometer. The top valley splitter occurs the intersection between the physical edge of graphene and the PN interface. The bottom valley splitter occurs at the intersection between the bottom side gate and the PN interface. (**B**) Valley-interferometer transmission $T_{\rm VI}$ as a function of B and V_1 for ν_2=0. $T_{\rm VI}$ oscillates in B with $\Delta B_{\rm MZ}\sim 83$ mT for ν_1=0 (black dashed line). (**C**) Valley-interferometer transmission $T_{\rm VI}$ as a function of B and V_2 for ν_1=-1. For $\nu_2 \leq$ -1 ($V_2 \leq 0.15$), $T_{\rm VI}$ oscillates in B with $\Delta B_{\rm MZ}\sim 34.5$ mT. The small period $\Delta V_{\rm VI}$ indicates that the interferometer area A is tuned by V_1. On top of these oscillations, we note oscillations that hardly depend on V_1 . For ν_1=0 highlighted by a black dashed line (V_2=0.55), fast oscillations in V_1 are washed out but oscillations we interpret to originate from an intermediate size interferometer persist.

500×500 nm^2 area), that can lead to intervalley scattering [4]. In particular, the spatial extension of the intervalley scattering induced by the defects can extend up to 15 nm away from the defects. We have already shown that intervalley scattering can occur at the intersection between the PN junction and the physical edge [5]. If the defect coincides now with the first intersection between the PN interface and the top (bottom) side gate (by sweeping the top and bottom gate voltages for example), intervalley scattering can also occur. Outside of these two additional intervalley scattering sites, edges states are separated by more than 80 nm. This leads to four possible intervalley scattering sites in total and three interferometer lengths.

Another possibility to explain the intervalley scattering could rely on the the valley properties of ν=0. At Landau level filling factor $\nu=$ 0, different phases are in competitions with distinct symmetry-breaking properties. Among these phases, one can cite the ferromagnet (F) state, the

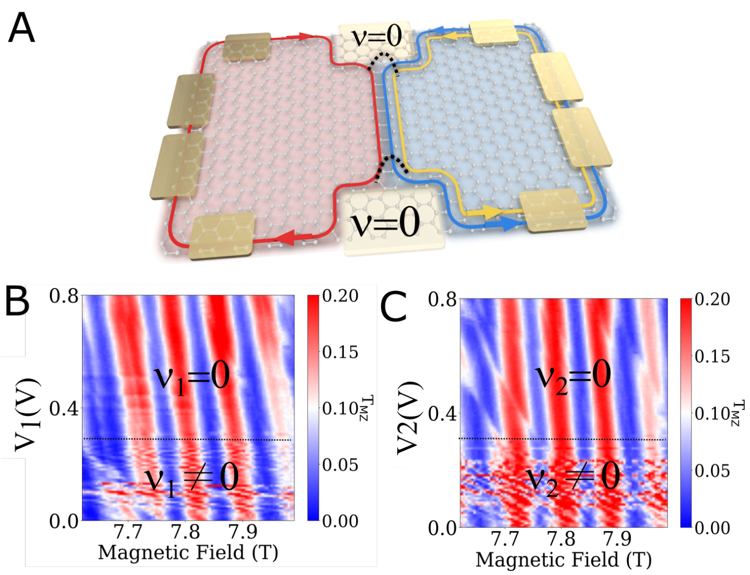

Supplementary Figure S 7. **Small length interferometer.** (**A**) Schematic representation of the small size interferometer. Top valley splitter occurs at the intersection between top side gate (ν_1=0) and the PN interface. The bottom valley splitter occurs at the intersection between the bottom side gate (ν_2=0) and the PN interface. (**B**) Valley-interferometer transmission $T_{\rm VI}$ as a function of B and V_2 for ν_1=0. $T_{\rm VI}$ oscillates in B with $\Delta B_{\rm MZ} \sim$ 81 mT for ν_2=0 (black dashed line). We interpret these ocillations to originate from a small size interferometer.

antiferromagnet (AF) state, or canted antiferromagnetic (CAF) state that can be adressed changing the ratio between the Zeeman energy and the Coulomb energy. Very little is known about interactions between propagating edge state and ν= 0 in the bulk. Our results could indicate that valley splitting can also occur along $\nu = 0$ in the bulk.

VI. DETERMINATION OF THE VISIBILITY AND ERROR BARS

The Aharonov-Bohm oscillations are fitted using the formula $\rm T_{MZ} = A \times \sin(2\pi\frac{B}{B_p} + \varphi) + C$ with A the oscillation amplitude, $\rm B_p$ the magnetic field period, φ an arbitrary phase offset and C the mean transmission value. The visibility is defined as $\rm Vis = \frac{T_{MZ,max} - T_{MZ,min}}{T_{MZ,max} + T_{MZ,min}}$. By replacing $\rm T_{MZ,max} = C + A$ and $\rm T_{MZ,min} = C - A$, the visibility can be written $\rm Vis = \frac{A}{C}$. The parameters A and C and their associated errors ΔA and ΔC respectively, are obtained from the fit. Then the error on the visibility is computed as $\rm \Delta Vis = \frac{C\Delta A - A\Delta C}{C^2}$.

VII. NOISE SETUP

A. Resonant RLC tank circuit

The voltage fluctuations of a RLC tank circuit is measured to extract the current noise of the sample. The RLC tank circuit is composed of the sample resistance Rs, in parallel with an inductor L (22 uF), a capacitor C (240 pF) and a resistor Rp (20 kohms) as shown in Supplementary Figure S8. Its resonance frequency is centered at 2.2 MHz. The capacitance C is mostly due to the distributed capacitance along the coaxial line connecting the sample to the cryogenic amplifier. About one meter of all the DC connections to the sample is wrapped with silver epoxy around the cold body of the fridge at 10mK in order to insure a low electronic temperature. The dissipation in the circuit is due to the DC resistance of the inductor (about 10ohm at room temperature), it is taken into account in the analysis by adding a resistance in series with the inductor. The resistance Rp is used to adjust the resonance bandwidth and to shunt the spurious signal coming from the 4K amplifier. Additional blocking capacitors are used to prevent the DC current from the sample to enter the RLC tank circuit.

B. Amplification chain

The voltage fluctuations of the RLC tank circuit is first amplified at 4K using a low noise amplifier, based on a commercial HEMT (ATF-34143), placed on the 4K plate of the dilution fridge, as shown in Supplementary Figure S8.

After a second stage of amplification at room temperature (amplifier NF SA-421F5), the signal is digitized with an acquisition card (ADLINK) to compute the noise spectral density.

In Supplementary Figure S9A, the voltage spectral density is shown for different temperature. Note that the voltage spectral density at 20 mK is subtracted from all curves to remove the noise background. Each curve is fitted to extract the gain of the amplification chain (G $\approx$ 800).

C. Base sample temperature

As shown in Supplementary Figure S9B, the current spectral density of the circuit is quasi-linear down to a temperature of 25 mK as described by the Johnson-Nyquist formula. Therefore, we consider that the sample electronic temperature equals the fridge temperature when no voltage drive is applied to it.

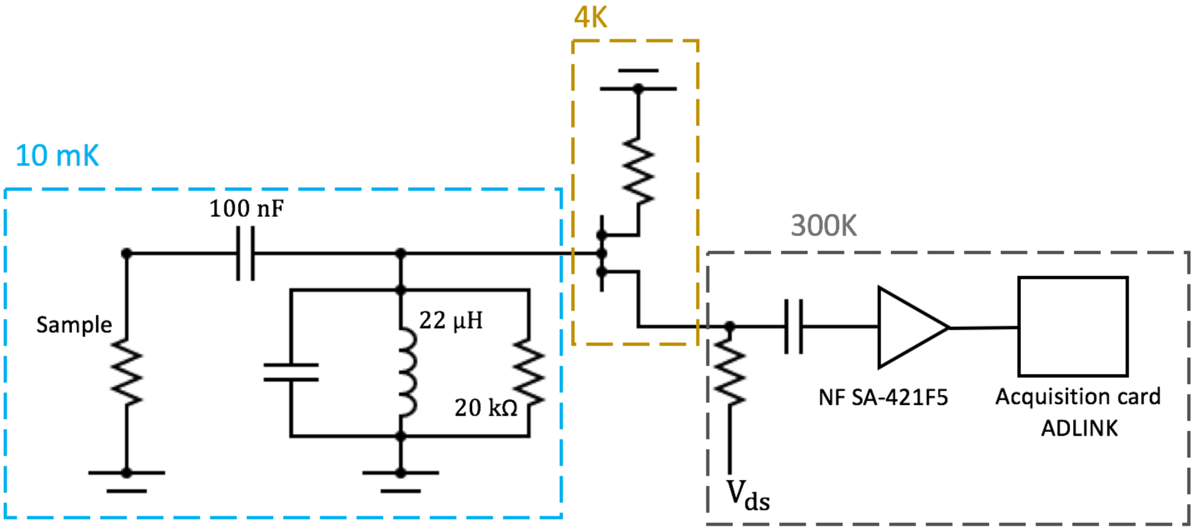

Supplementary Figure S 8. **Noise setup.** Schematic representation of the noise experimental set up. A RLC tank circuit is connected to the sample at 10 mK. The capacitance of the tank circuit is mainly due to the coaxial cable connecting the sample to the gate of the HEMT (ATF 34-143) at 4K. The noise signal is further amplified (NF SA-421F5) and digitized (ADLINK) at room temperature.

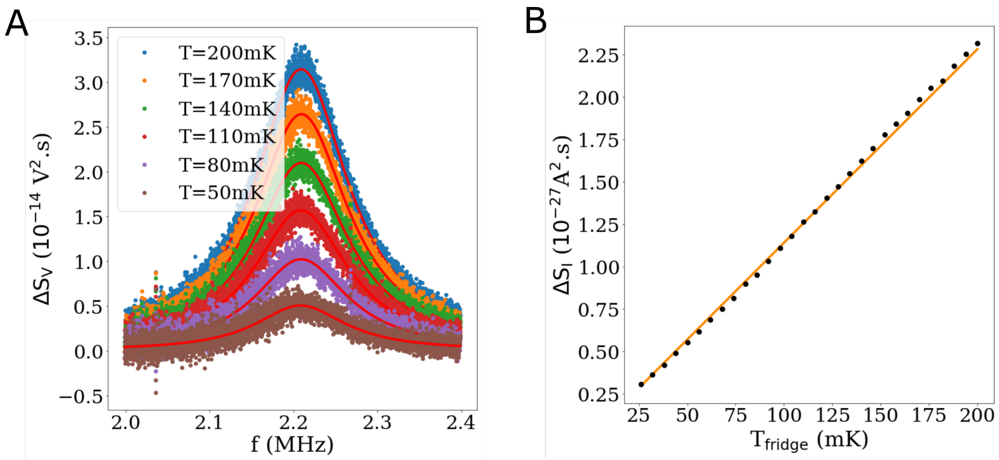

Supplementary Figure S 9. **Calibration of the gain of the amplification chain**

A: Voltage spectral density as a function of frequency for different temperature after amplification. The red curves are the fits of the Johnson-Nyquist noise of the circuit which is composed of the sample resistance in parallel with a RLC resonator. The gain of the amplification chain is obtained from the fit.

B: Current spectral density of the circuit as a function of temperature.

VIII. SHOT NOISE OF THE BEAM SPLITTERS

Using the Landauer-Buttiker formalism, the excess current spectral density induced by the partitioning of several modes through a scatterer is given by:

$$\Delta \mathrm{S_I} = \frac{2\mathrm{e}^2}{\mathrm{h}} \sum_{\mathrm{n}} \mathrm{D_n}(1 - \mathrm{D_n}) \left[\mathrm{eV_{dc}} \coth\left(\frac{\mathrm{eV_{dc}}}{2\mathrm{k_B T}} \right) - 2\mathrm{k_B T} \right] \quad (1)$$

where n is the number of electronic channels, $\mathrm{D_n}$ is their transmission, $\mathrm{V_{dc}}$ is the voltage bias, and T is the electronic temperature.

In the quantum Hall regime, the number of channel is tuned by the filling factor and the transmission on both edge of a pn junction can be adjusted using side gates. In Supplementary Figure S10AB the transmission D of the upper edge of the pn interface is adjusted to D=0.39 using the top side gate while the mixing on the bottom edge is cancelled (D=0) by fixing the bottom side gate value to V=-0.5V. The shot noise shown in Supplementary Figure S10AC is in agreement with the scattering theory for two channels. This confirms unambiguously that both channels are mixing at the edge of the pn junction.

IX. MEASUREMENT OF THE ELECTRONIC TEMPERATURE

Accurate measurement of the electronic temperature is done using Johnson–Nyquist noise that relies on the quantification of the resistance at ν=2 and the temperature of the system. Current noise of the Hall resistance is given by:

$$S_I = \frac{4k_B T_{el}}{R_H} \quad (2)$$

with T_{el} the electronic temperature, $R_H = h/2e^2$ the Hall resistance at $\nu = 2$ and k_B the Boltzmann constant. For the noise measurement, the current noise is converted into voltage fluctuations across a RLC circuit (300 kHz bandwidth for a resonant frequency of 2.2 MHz) combined with a homemade cryogenic amplifier(see in Supplementary Figure S11A). After further amplification and digitization, the autocorrelation voltage noise spectra is calculated in real-time by a computer. We measure the Johnson-Nyquist noise of the Hall resistance as a function of the temperature of the fridge from 20 mK to 200 mK. The linear dependence of the Johnson-Nyquist noise as a function of the temperature on the whole range confirms that electrons are correctly thermalized and T_{el}=20mK at the base temperature of the fridge.

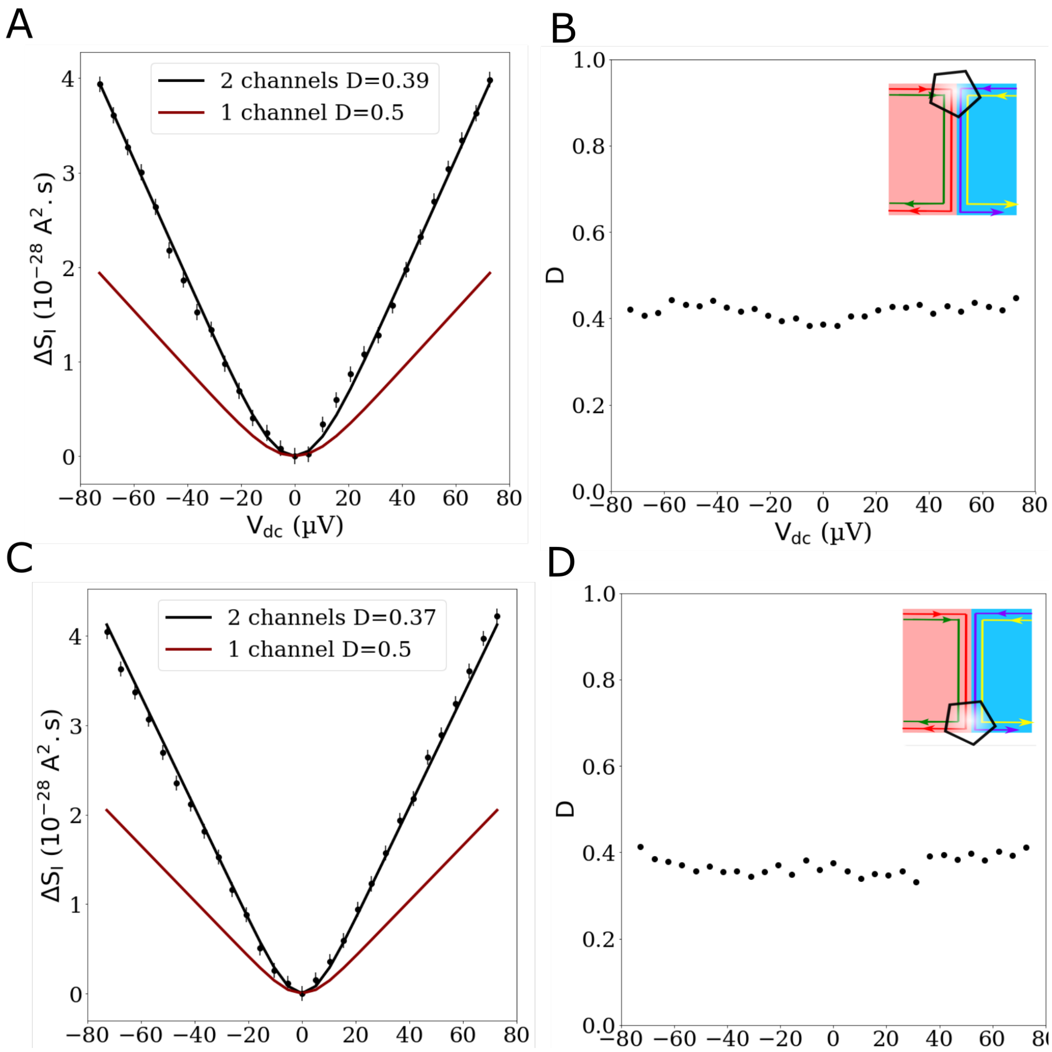

Supplementary Figure S 10. **Shot noise of the two bean splitters.**

A: Current spectral density as a function of DC voltage. The transmission measured (B) is in good agreement with the transmission obtained by fitting the noise with the scattering formula for two channels. Red solid line shows the maximum shot noise obtained from a single channel which can not account for the experiment. The bottom side gate voltage is fixed at -0.5V to avoid the mixing at the bottom beam splitter. All error bars are the s.e.m. calculated from the auto-correlation noise spectrum standard deviation. **B**: Transmission obtained from the current measured simultaneously with (A). **C**: Current spectral density as a function of DC voltage. The transmission measured (D) is in good agreement with the transmission obtained by fitting the noise with the scattering formula for two channels. Red solid line shows the maximum shot noise obtained from a single channel which can not account for the experiment. The top side gate voltage is fixed at -0.5V to avoid the mixing at the top beam splitter. **D**: Transmission obtained from the current measured simultaneously with (C).

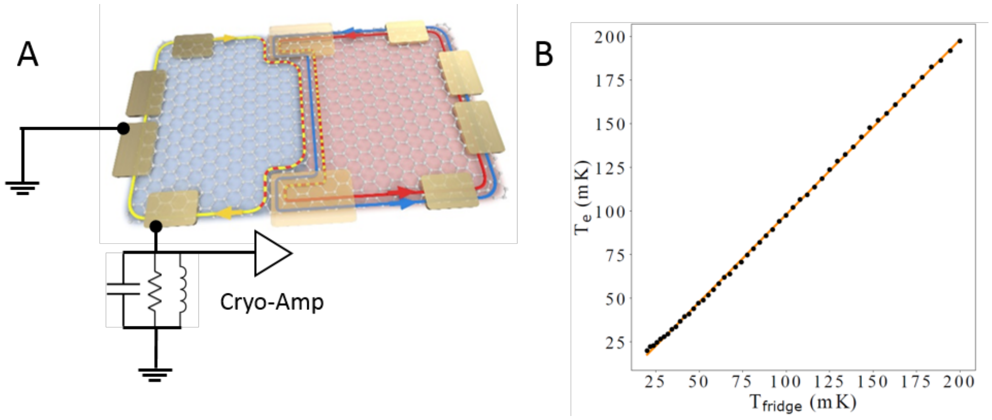

Supplementary Figure S 11. **Shot noise measurements.** (**A**) Schematic representation of the shot noise experimental set up. Current fluctuations of the Hall resistance are converted into voltage fluctuations across a RLC circuit further amplifed by a cryogenics amplifier (**B**) T_{el} extracted from the Johnson-Nyquist noise measurement as a function of the fridge temperature. The linear behavior over the whole temperature range confirms that electrons are correctly thermalized to the fridge.

X. 1/F NOISE AND DECOHERENCE

The 1/f noise is linked to statistical fluctuations due to some inhomogenities or defects trapped in the conductor. The defect will absorb or emit electrons. If the defect is close enough to one arm of the interferometer, this can lead to dephasing or decoherence. It can also change the resistance of the sample either by changing the electronic density or by modifying the electrostatic potential seen by the conducting electrons. The power spectral density associated with a single defect is given by:

$$S_N(\omega) = 4\overline{\Delta N^2}\frac{\tau}{1+\omega^2\tau^2} \tag{3}$$

With the typical time scale the defect absorb or emit electrons and ΔN is the fluctuation of the involved charges. This noise is also called telegraphic noise since it involves two-level systems: the electron is trapped or not and the power spectral density has a Lorentzian shape. The 1/f is obtained when several defects with different time scales τ_i are involved. If we assume that τ follows an thermally activated law $1/\tau \propto e^{-E/k_B\theta}$ with E the height of the potential barrier and θ the temperature, and if the distribution of E is uniform on an interval $[E_{min}, E_{max}]$, averaging over all the defects gives a power spectral density:

$$S_N(f) = \frac{1}{2\pi f}\frac{k_B\theta}{E_{max}-E_{min}}(arctan(\omega\tau_{max}) - arctan(\omega\tau_{min})) \tag{4}$$

If there is 1/f noise in our sample, we should detect it by measuring the noise as a function of the temperature. In Supplementary Figure S9, the power spectral density is measured for different temperatures. At large temperature (200mK), we do not observe any enhancement of the noise a lower frequency.

To further confirm it, we have measured the shot noise of a valley splitter as a function of a given transmission. Supplementary Fig S10 shows the excess shot noise defined as ΔS_I (V_{DC})=$S_I(V_{DC})-S_I(0)$ as a function of the applied bias V_{DC} for a transmission D=0.5. We compare $\Delta S_I(\mathrm{V}_{DC})$ to the expected shot noise auto-correlation: $\Delta S_I(\mathrm{V}_{DC})=\frac{2e^2}{h}\sum_n D_n(1-D_n)[eV_{DC}coth(\frac{eV_{DC}}{2k_BT})-2k_BT]]$. In the present case $\sum_n D_n(1-D_n)$=2D(1-D) for the two partitioned modes (two pseudo-spin). The agreement with the expected shot noise confirms the absence of 1/f noise that would be induced by defects close to the valley splitter.

XI. THEORETICAL MODELS

We describe the theoretical models for the decoherence of the graphene MZI, focusing on the filling factor of $\nu_P = -1, \nu_N = 2$. Two dephaisng mechanisms are mainly considered, (A) a capacitive intra-edge interaction model [6, 8–10], and (B) a short-range inter-edge interaction model [11].

A. Capacitive intra-edge interaction

For the sake of self-containment, we here repeat the introduction of the intra-edge capacitive interaction model, given in the Methods, whose Hamiltonian is described by

$$\mathcal{H} = \sum_{\alpha=r,l}[\mathcal{H}_{\alpha,0} + \mathcal{H}_{\alpha,\mathrm{intra}}] + \mathcal{H}_T. \quad (5)$$

Here, $\mathcal{H}_{\alpha,0} = -\hbar v \int dx \psi_\alpha^\dagger(x) i\partial_x \psi_\alpha(x)$ is the non-interacting Hamiltonian for the left ($\alpha = l$) and right ($\alpha = r$) arm of the MZI, and $\mathcal{H}_{\alpha,\mathrm{intra}} = \frac{1}{2}\frac{gv\hbar}{L}(\int_0^L \rho_\alpha(x)dx - N_{g,\alpha})^2$ accounts for the capacitive coupling of each arm with the gates, corresponding to the Eq. (1) of the main text. v is the drift velocity, L is the length of the arms, $\rho_\alpha(x) =: \psi_\alpha^\dagger(x)\psi_\alpha(x)$: is the (normal-ordered) electron density of arm α, $\psi_\alpha^\dagger(x)$ creates an electron at position x of arm α, $N_{g,\alpha}$ is tuned by the gate voltage, and g is the dimensionless interaction strength. The total charge $Q_\alpha = \int_0^L \rho_\alpha(x)dx$ inside each arm is interacting capacitively. The beam splitters of the MZI at the upper and lower side gates are described by $\mathcal{H}_T = \mathcal{T}_U + \mathcal{T}_D + h.c.$, where $\mathcal{T}_U(t) = \hbar v t_U \psi_r^\dagger(0,t)\psi_l(0,t)$ and

$\mathcal{T}_D(t) = \hbar v t_D \psi_r^\dagger(L,t)\psi_l(L,t)$. Employing the Peierls substitution of $t_U \to e^{ieV_{\rm DC}t/\hbar}e^{i\phi_{\rm AB}/2}t_U, t_D \to e^{ieV_{\rm DC}t/\hbar}e^{-i\phi_{\rm AB}/2}t_D$, we account the bias voltage $V_{\rm DC}$ and the Aharonov-Bohm phase $\phi_{\rm AB}$ enclosed by the MZI loop.

To calculate the thermal decay of the MZI visibility, we consider the tunneling regime of small t_U and t_D. In this case, the visibility Vis of the differential conductance at the zero bias limit is given by

$$\text{Vis} = \frac{2|t_U|\cdot|t_D|}{|t_U|^2+|t_D|^2}\left|2\pi v^2 \sum_{\eta=\pm}\eta\int dt\, it G^\eta(t)\right|. \tag{6}$$

Here, $G^\eta(t)$ is an electron Green's function at finite temperature under the Hamiltonian $\sum_{\alpha=l,r}[\mathcal{H}_{\alpha,0}+\mathcal{H}_{\alpha,\rm int}]$, $G^+(t) = \langle\psi_l^\dagger(L,t)\psi_l(0,0)\rangle\langle\psi_r(L,t)\psi_r^\dagger(0,0)\rangle$, and $G^-(t) = \langle\psi_l(0,0)\psi_l^\dagger(L,t)\rangle\langle\psi_r^\dagger(0,0)\psi_r(L,t)\rangle$. They are obtained using the bosonization technique,

$$\begin{aligned} G^\eta(t) =& \frac{\exp(\delta\mathcal{G}^\eta(t))}{(\frac{2v}{k_BT}\sin[\frac{\pi k_B T}{v}(a-i\eta(L-vt))])^2}, \\ \delta\mathcal{G}^\eta(t) =& i2\eta L\int dq \frac{\frac{g}{2\pi}(\frac{\sin qL/2}{qL/2})^2}{1+\frac{g}{2\pi}\frac{\sin qL/2}{qL/2}e^{-i\eta qL/2}}\frac{e^{i\eta q(L-vt)}}{1-e^{-qv/k_BT}} + i\frac{g}{1+\frac{g}{2\pi}}(N_{g,l}-N_{g,r}), \end{aligned} \tag{7}$$

where $a > 0$ is the infinitesimal short-distance cutoff, and $\delta\mathcal{G}^\eta(t)$ is from the capacitive coupling. We note that $N_{g,\alpha}$ does not affect the visibilities (it contributes only to the phase shift of the Aharonov-Bohm oscillations).

The visibility shows the scaling behavior of $\text{Vis}(L,T) = \text{Vis}(LT)$, which is expected in our experiments. This can be seen from the fact that Eqs. (6) and (7) are invariant under the rescaling of the variables $L \to bL$, $T \to b^{-1}T$, $t \to bt$, $a \to ba$, $q \to b^{-1}q$, with a rescaling parameter $b > 0$.

We calculate Eq. (6) numerically, and find that the result with the parameters of $g = 3.3$ and $v = 4.4\times10^4 m/s$ fit the data well. To gain insight of the decoherence by the capacitive intra-edge interaction, we plot the result for various values of the interaction strength g [see Supplementary Figure S12]. For small g, the visibility decreases as g increases. However for g larger than a certain value ($\sim$ 10), the visibility becomes larger as g increases. This is reminiscent of the visibility recovery for the strong capacitive interaction, as predicted in Ref. [8]. It would be interesting to experimentally verify this behavior by tuning the strength of the capacitive coupling (e.g., by controlling the distance between gates and the graphene sheet).

One can observe that the crossover occurs near the temperature of $T \simeq \hbar v/k_BL$, and does not depend much on g. Physically, when an electron enters to the interferometer arm, the capacitive interaction makes the fluctuation of the charge densities and provides which path information.

When the thermal width of the electron wavepacket is larger than the interferometer arm lengths, the fluctuation, and the resulting dephasing is suppressed.

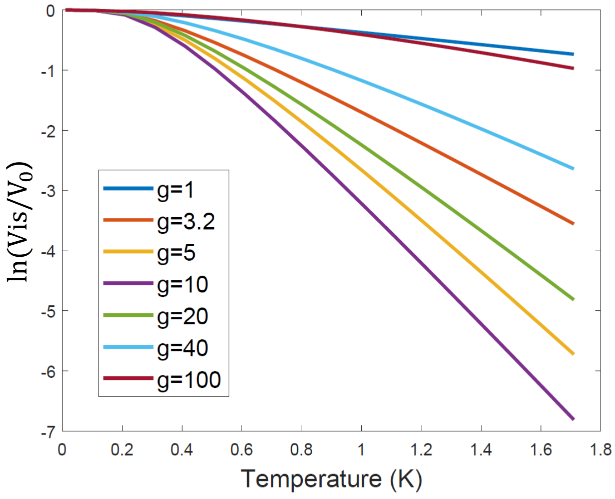

Supplementary Figure S 12. Visibiliy decay as a function of temperature for various values of the interaction strength g of the capacitive intra-edge interaction model. The drift velocity $v = 4.4 \times 10^4 m/s$ and the arm length $L = 1.5 \mu m$ are used.

To calculate the source-drain bias voltage dependence of the visibility, we employ the method developed in Ref. [10]. In this case, we consider the non-perturbative regime of t_U and t_D in which the transmission probability of the beam splitters $\in [0, 1]$. The bias voltage dependence of the visibility, called the lobe pattern, has the feature that the relative amplitude of the side lobe increases as the transmission probability of the first beam splitter deviates from 1/2. The calculation results are in good agreement with the experimental data, as shown in Supplementary Figure S13. For better qualitative agreement, we additionally multiply the phenomenological factor [12] of $\exp\left(-V_{\mathrm{DC}}^2/2V_0^2\right)$ with $V_0 = 0.11$ mV to the interference current.

B. Dependence of lobe pattern on beam splitting probability and comparison with the intra-channel interaction model

Our intra-edge interactions model qualitatively reproduces the lobe pattern as a function of the transmission as shown in Supplementary Figure S13.

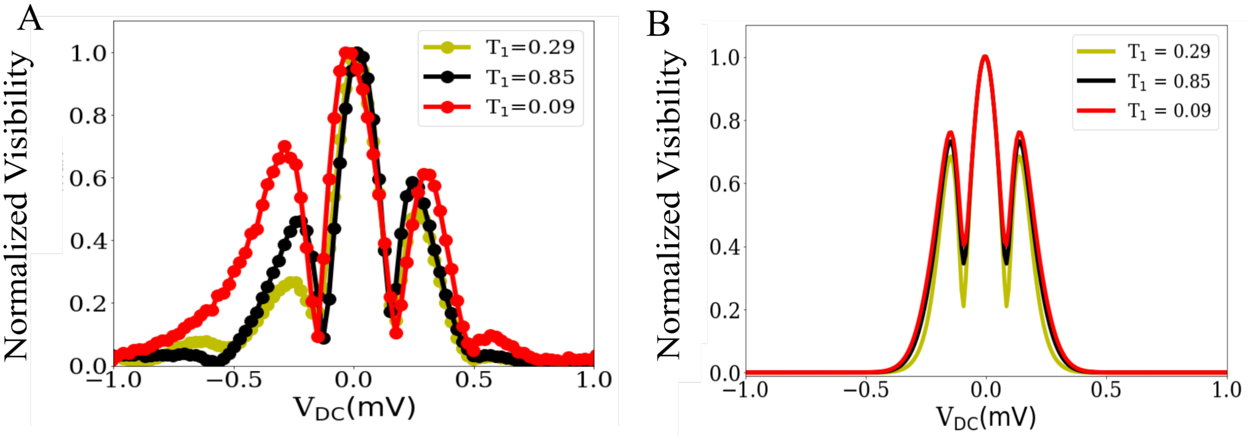

Supplementary Figure S 13. Dependence of lobe pattern on beam splitting probability. (A) Normalized measured visibility as a function of the DC bias V_{DC} for different values of the transmission probability T_1 of the top beam splitter. It is measured with the large interferometer at 9T and (ν_N,ν_P) = (2,-1) by applying V_{DC} to the upper right ohmic contact. (B) Normalized visibility, computed with the intra-channel interaction model. We use the parameters chosen in Fig. 2(B), and multiply, to the interference current, the phenomenological factor [12] of exp(-$V_{\mathrm{DC}}^2/2V_0^2$) with V_0=0.11 mV that can describe voltage fluctuations at the source.

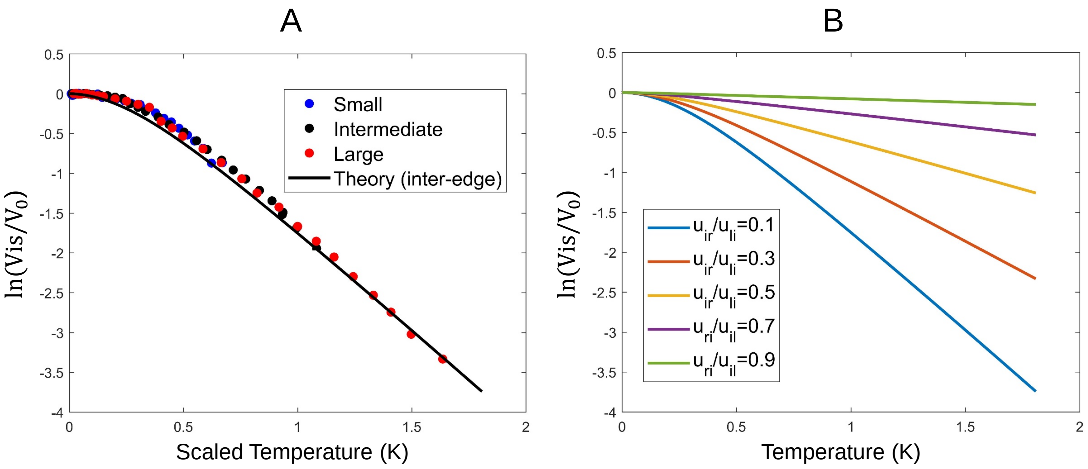

Supplementary Figure S 14. Visibility as a function of temperture. (A) The best fit with the experimental data. The parameters of $v_r = v_l = v_i = 1.35 \times 10^5 m/s, u_{li} = 6.8 \times 10^4 m/s, u_{ir} = 6.8 \times 10^3 m/s$ are used, but the large asymmetry between the interaction strengths ($u_{ir}/u_{li} = 0.1$) are unlikely in our experiment. (B) The dependence of the visibility on the interaction strength asymmetry. All the parameters except u_{ir} are same with the case (A). When the interaction strength is completely symmetric, i.e. $u_{ir} = u_{li}$, there is no decoherence at all.

C. Short-range inter-edge interaction

Here we consider decoherence in the short-range inter-edge interaction model, focusing on the filling factors $\nu_P = -1, \nu_N = 2$. The short-range interactions (see $\mathcal{H}_{\text{inter}}$ below) between the adjacent edge channels along the PN interface of our experimental setup are taken into account as

$$\mathcal{H} = \sum_{\alpha=r,i,l} \mathcal{H}_{\alpha,0} + \mathcal{H}_{\text{inter}} + \mathcal{H}_T, \quad \mathcal{H}_{\text{inter}} = \hbar\pi \sum_{\alpha,\beta} u_{\alpha\beta} \int dx \rho_\alpha(x)\rho_\beta(x). \tag{8}$$

The $\alpha = r, l$ channels are the right and left interfering MZI arms as described above, and $\alpha = i$ channel describes the intermediate edge channel having the spin opposite to the spin of the MZI arms (hence this channel does not participate in the MZI in the non-interacting limit). Along the PN interface, this channel $\alpha = i$ is in the N side of $\nu = 2$ and it is expected to be sandwiched by the two MZI arms $\alpha = r, l$. The diagonal strength $u_{\alpha\alpha}$ of the inter-edge interaction renormalizes the velocity of the channel α, $v_\alpha = v \to v + u_{\alpha\alpha}$, while the off-diagonal strength $u_{\alpha\beta}$ with $\alpha \neq \beta$ may fractionalize electrons as in the quantum Hall edge channels at filling factor $\nu = 2$ of GaAs 2DEG samples. We compute the visibility of the MZI differential conductance, following Ref. [11].

However, the unique edge structure of our graphene MZI largely suppresses the resulting decoherence. In contrast to the GaAs 2DEG samples whose two interfering edge channels differently couple to other edge channels, the interfering edges $\alpha = r, l$ in our sample interact with the common adjacent edge $\alpha = i$. If the interaction strengths are symmetric, the short-range inter-edge interaction cannot induce neither fractionalization nor decoherence. While one can force the asymmetry to fit the experimental visibility decay as a function of temperature [Supplementary Figure S14], the interaction strength u_{li} between the left MZI arm and the intermediate edge channel should be about 10 times larger to the one u_{ir} between intermediate edge channel and the right arm of the MZI, which is not adequate to our sample. In addition, the theory based on the inter-edge interaction provides a faster dephasing compared to the experiments in the low temperature regime. We remark that in the case of $(\nu_N, \nu_P) = (2, -2)$, the symmetric edge alignments between two MZI arms are broken, so the interaction can induce a more dephasing, which is consistent with the Fig.3 of the main text.

Also, the qualitative features of the bias voltage dependence in Supplementary Figure S15, however, is apparently different from the experimental data. In the experiment, the lobe structure is almost independent of whether electrons are injected to the MZI from the P side of $\nu = -1$ (by applying the source-drain bias voltage to the single edge channel of the P side) or the N side of $\nu = 2$ (by applying the voltage to the two edge channels of the N side); see Fig. 4 of the main

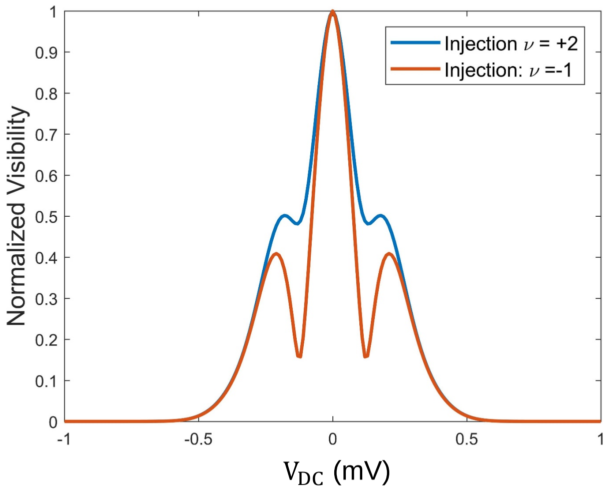

Supplementary Figure S 15. The lobe pattern by the inter-edge interactions. We use parameters chosen in Figure S14 and in Fig. 2(B), and multiply, to the interference current, the phenomenological factor [8] of $\exp(-V_{\mathrm{DC}}^2/2V_0^2)$ with $V_0 = 0.15mV$ that can describe voltage fluctuation at the source.

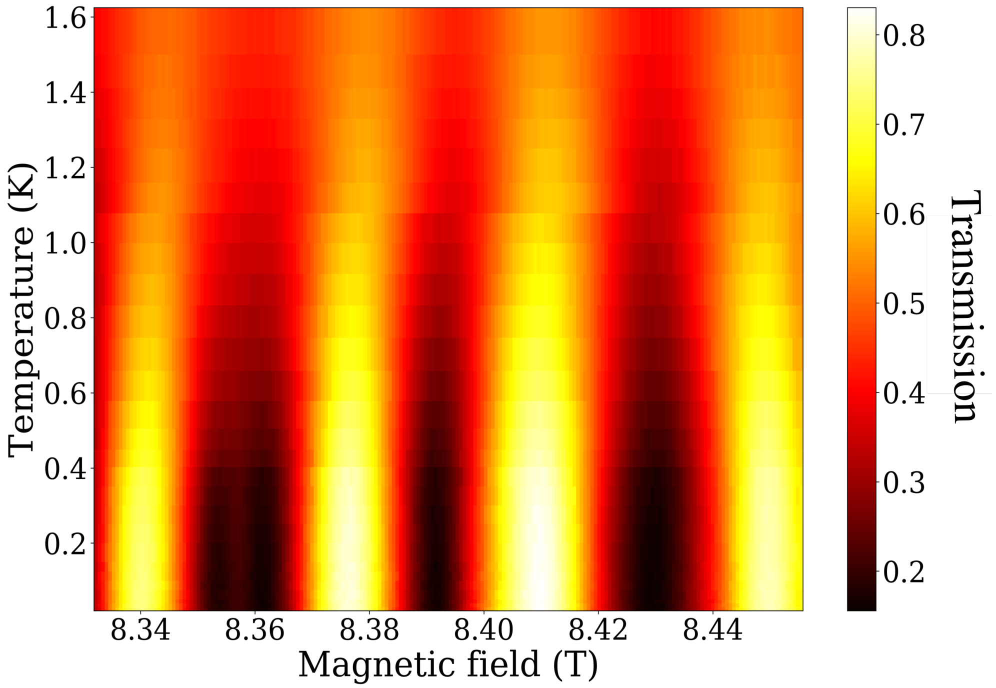

Supplementary Figure S 16. Mach-Zehnder transmission as a function of the magnetic field and the temperature for the intermediate interferometer.

text. But the calculation result in Supplementary Figure S15 does depend on the injection side. This asymmetric feature with respect to the injection sides generally happens in the short-range inter-edge interaction model, because the occupation of the zero mode of the PN interface channels

(hence the dynamical phase gain from their inter-edge interaction) depends on the injection side. A similar effect was reported [11] in a GaAs MZI at filling factor $\nu = 2$, where the case of a bias voltage applied only to the edge channel forming the MZI is compared with the case of the voltage applied to both the two edge channels. Therefore, the inter-edge interaction model cannot explain our experimental data, and it would not be a main source of the decoherence of our MZI.

XII. AHARONOV-BOHM OSCILLATIONS AS A FUNCTION OF THE TEMPERATURE

As explained in the main text, the irrelevance of the inter-edge interaction is attributed to the geometry of our experimental setup. As the distance between the graphene and the electrostatic gates is much shorter than the distance between the channels ($> 100nm$) of the PN interface, the inter-edge interaction would be screened out and subdominant. This makes a stark difference with the MZIs formed in GaAs 2DEG samples.

SUPPLEMENTARY REFERENCES

---

[1] F. Pizzocchero et al., Nat Commun 7, 11894 (2016)

[2] L. Wang et al., Science 342, 614-–617 (2013)

[3] Déprez, C. et al., A tunable Fabry-Pérot quantum Hall interferometer in graphene, Nature Nanotechnology 16, 555 (2021)

[4] Joucken, F. et al., Direct Visualization of Native Defects in Graphite and Their Effect on the Electronic Properties of Bernal-Stacked Bilayer Graphene, arXiv:2104.10620 (2021)

[5] Jo, M. et al., Quantum Hall Valley Splitters and a Tunable Mach-Zehnder Interferometer in Graphene, Phys. Rev. Lett. 126, 146803 (2021).

[6] S.-C. Youn, H.-W. Lee, and H.-S. Sim, Nonequilibrium Dephasing in an Electronic Mach-Zehnder Interferometer, Phys. Rev. Lett. **100**, 196807 (2008).

[7] Flor, I.M. et al., How do edge states position themselves in a quantum Hall graphene pn junction?, arXiv:2201.12025 (2022).

[8] S.-Y. Lee, H.-W. Lee, and H.-S. Sim, Visibility recovery by strong interaction in an electronic Mach-Zehnder interferometer, Phys. Rev. B **86**, 235444 (2012).

[9] D. L. Kovrizhin and J. T. Chalker, Exactly solved model for an electronic Mach-Zehnder interferometer, Phys. Rev. B **80**, 161306(R) (2009).

[10] D. L. Kovrizhin and J. T. Chalker, Multiparticle interference in electronic Mach-Zehnder interferometers, Phys. Rev. B **81**, 155318 (2010).

[11] I. P. Levkivskyi and E. V. Sukhorukov, Dephasing in the electronic Mach-Zehnder interferometer at filling factor $\nu = 2$, Phys. Rev. B **78**, 045322 (2008).

[12] P. Roulleau, F. Portier, D. C. Glattli, P. Roche, A. Cavanna, G. Faini, U. Gennser, and D. Mailly, Finite bias visibility of the electronic Mach-Zehnder interferometer, Phys. Rev. B **76**, 161309(R) (2007).
